# Integrative assessment of brain and bone invasion in meningioma patients

**DOI:** 10.1186/s13014-019-1341-x

**Published:** 2019-07-29

**Authors:** Kerstin Zwirner, Frank Paulsen, Jens Schittenhelm, Irina Gepfner-Tuma, Ghazaleh Tabatabai, Felix Behling, Marco Skardelly, Benjamin Bender, Daniel Zips, Franziska Eckert

**Affiliations:** 1 Department of Radiation Oncology, University Hospital Tuebingen, Hoppe-Seyler-Str. 3, 72076 Tuebingen, Germany; 2 Department of Neuropathology, Institute of Pathology and Neuropathology, University Hospital Tuebingen, Calwerstr. 3, 72076 Tuebingen, Germany; 3 Interdisciplinary Division of Neuro-Oncology, Departments of Neurology and Neurosurgery, University Hospital Tuebingen, Hoppe-Seyler-Str. 3, 72076 Tuebingen, Germany; 4 Department of Neurosurgery, University Hospital Tuebingen, Hoppe-Seyler-Str. 3, 72076 Tuebingen, Germany; 5 Diagnostic and Interventional Neuroradiology, Department of Radiology, University Hospital Tuebingen, Hoppe-Seyler-Str. 3, 72076 Tuebingen, Germany; 6 Center for CNS Tumors, Comprehensive Cancer Center Tuebingen, Hoppe-Seyler-Str. 3, 72076 Tuebingen, Germany; 7 German Cancer Consortium (DKTK), German Cancer Research Center (DKFZ) partner site Tuebingen, Hoppe-Seyler-Str. 3, 72076 Tuebingen, Germany

**Keywords:** Radiotherapy, Meningioma, Brain invasion, Bone involvement, Prognostic factors

## Abstract

**Background:**

Various prognostic factors have been suggested in meningioma patients including WHO grading, brain invasion and bone involvement, for instance. Brain invasion was included as an independent criterion in the recent WHO classification. However, assessability of brain or bone involvement is often limited or varies between histopathologic, operative and imaging reports. Objective of our study was to investigate prognostic values including brain and bone involvement according to different clinical approaches.

**Methods:**

A cohort of 111 patients was treated with primary, adjuvant or salvage irradiation between 2008 and 2017 using intensity-modulated radiotherapy. Positron-emission tomography (PET) was available for treatment planning in 81% of patients. Clinical data were extracted from the medical reports. Brain and bone involvement were stratified separately according to histopathologic, operative and imaging reports as well as judged in synopsis.

**Results:**

WHO grade I tumours, lower estimated proliferation index, primary versus recurrence treatment and localization (i.e. skull base, optic nerve sheath) were beneficial prognostic factors for local control. Judgement of brain and bone invasion partly differed between diagnostic modalities. In synopsis, brain or bone invasion did not show a significant influence on local control rates.

**Conclusions:**

Several previously described prognostic factors could be reproduced. However, partly divergent histopathological, surgical and image-based judgements could be found in regard to brain and bone invasion and all methods imply limitations. Therefore, we suggest a particular, complemental synopsis judgement. In synopsis, brain or bone involvement did not coherently impair local control in our irradiated patients. This might be explained by elaborate radiation techniques and PET-based treatment planning.

**Electronic supplementary material:**

The online version of this article (10.1186/s13014-019-1341-x) contains supplementary material, which is available to authorized users.

## Background

Primary radiotherapy or surgical resection are considered as treatment options for patients with progressive or symptomatic meningiomas [1]. Furthermore, postoperative radiotherapy is indicated if patients are at risk for local failure. Previously described risk factors for recurrence imply incomplete resection [2], higher histopathological grading according to the World Health Organization (WHO) classification [3], increased proliferation activity (e.g. Molecular Immunology Borstel (MIB-1) immunohistochemistry) [4], bone involvement [5] and brain invasion [6]. Histological brain invasion is even considered as an individual criterion for atypia (WHO grade II) and was included in the recent WHO classification 2016 [7]. Involvement of brain tissue is commonly defined by histopathological criteria referred to Perry et al. (1997) [6]. However, in this study, the majority of meningioma specimens lacked central nervous system (CNS) tissue and therefore, brain invasion could not be determined. The lack of brain tissue might be due to partial resections or because the arachnoid layer was respected by the meningioma and a resection of brain tissue was not considered necessary during surgery. The authors considered these samples (85%) as (pathologically) ‘unassessable’ [6]. In regard to the ‘assessable’ (i.e. adjacent CNS tissue; invasion determined as present or absent) specimens, a considerable discrepancy between surgeon’s gross impression and histologic findings was claimed (κ = 0.09; poor agreement) [6]. Therefore, clinicopathological judgement of brain invasion implies several pitfalls. Furthermore, many risk factors were mainly established in surgically treated cohorts and translation to irradiated patients remains controversial. Even in surgically treated patients, the stand-alone impact of brain invasion on prognosis has been critically addressed, recently [8–10]. Also, judgement of bone involvement is sometimes ambiguous, even though it has considerable implications on target volume definition in radiation treatment planning [11].

The aim of our study was to validate previously described prognostic factors in patients treated with radiotherapy. Furthermore, we evaluated histopathological, operative and imaging reports individually and in synopsis in regard to brain and bone invasion to consider potential discrepancies and limitations of the individual modalities.

## Methods

In our single-centre cohort of meningioma patients, we retrospectively screened for patients treated with intensity-modulated radiotherapy (IMRT) with or without prior surgery between 2008 and 2017. The analysis was approved by the local ethics committee (417/2017B02). Patients without evaluable follow-up or re-irradiated patients were excluded. Clinical features and follow-up data were extracted from the medical reports.

The extent of resection (subtotal versus gross tumour resection) was documented according to the operative report and post-operative imaging. The localization of meningiomas was subclassified into optic nerve sheath and skull base meningiomas versus ‘other’ meningiomas. Furthermore, the availability of positron-emission tomography (PET) with somatostatin-analogue tracers (combined with computed tomography (PET/CT) or magnet resonance imaging (PET/MRI)) for treatment planning and the intention of irradiation (primary radiotherapy, adjuvant radiotherapy, adjuvant radiotherapy after salvage surgery or salvage radiotherapy) were recorded. Salvage therapies were defined as therapy initiation for tumour recurrence or progression.

All patients in our series were treated with IMRT and image guidance as fractionated stereotactic radiotherapy. The applied radiation dose ranged between 54.0Gy (WHO grade I meningiomas, optic nerve sheath and skull base meningiomas without prior surgery) and 60.0Gy (WHO grade II meningiomas in other localizations). In case of meningiomas with close proximity to the optic nerve or chiasm, patients were treated with a simultaneous integrated boost (1.7Gy / 1.8Gy) up to a total dose of 51.0Gy / 54.0Gy (planning target volumes PTV51 / PTV54). The gross tumour volume (GTV) was defined as the contrast-enhancing region in T1-weighted MRI in synopsis with the PET dataset, both co-registered to the planning CT. An obvious dural tail was included within the GTV. For skull base meningiomas near the visual pathway or the brain stem, the PTV was defined as GTV plus a safety margin: 5 mm margin for the PTV51 and 2 mm margin for the PTV54. For other tumours, larger margins of approximately 10 mm alongside the meninges were used for the clinical target volume (CTV) and 5 mm safety margin to the PTV. In case of brain or bone involvement, approximately 10 mm around the cavity into the brain / bone or around the GTV were included as also described as accepted treatment strategies in recent reports [12, 13].

Most histopathological reports for grading were provided before the recent 2016 WHO classification was established. However, all samples with histologically proven brain invasion (which was recorded independently) from this time period were also classified as WHO grade II meningiomas because further criteria of atypia (mitotic activity) were apparent. Thus, all samples are consistent with the latest WHO classification (2016). The majority of histopathologic reports provided estimated MIB-1 staining to indicate the proliferative activity. The average values of the reports were documented and patients were arbitrarily separated into two risk groups according to the median (MIB-1 < 4% and MIB-1 ≥ 4%) based on previous reports [14].

Brain and bone invasion were retrospectively assessed based on histopathological, operative and diagnostic imaging reports separately and in synopsis. Assessable data of the different modalities that allowed a distinct consideration (‘yes’ or ‘no’) varied and are shown in the Additional file 1: Figures S1 and S2. The graduation of Perry et al. (1997) [6] was used to record histopathological brain invasion. If no CNS parenchyma was reported, the sample was regarded as histopathologically unassessable. In case of adjacent CNS tissue, brain invasion was considered as either present or absent.

**Fig. 1:**
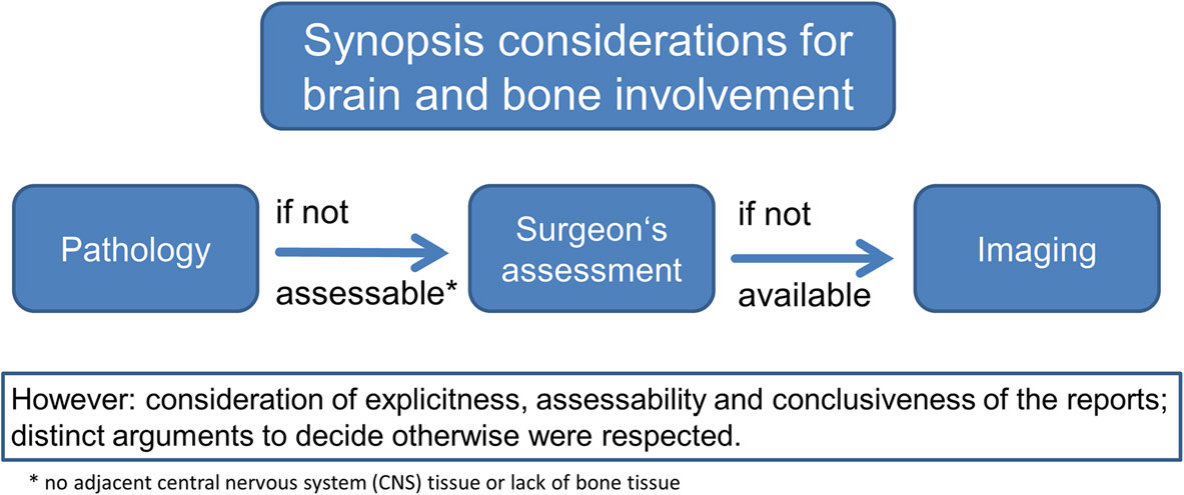
Synopsis evaluation considerations

For the synopsis evaluation, all information was considered (Fig. 1). If data of one modality was missing and others commented on invasion, the available information was used to compensate the missing ones. In case of contradicting information, plausibility was considered by the explicitness of the reports. If there were no distinct arguments to decide otherwise, pathologic reports were ranked as most important, followed by the surgeon’s description. In synopsis, invasion was considered as absent if none of the reports commented on involvement. Therefore, in synopsis evaluation, all patients of the cohort were included.

Progression-free survival (PFS) was calculated from the first day of radiotherapy and defined by local control of the irradiated lesion according to the last follow-up. For the statistical analysis we used SPSS (version 25, IBM Corp., Armonk, NY). To estimate local recurrence, the Kaplan-Meier method was applied. For comparison of different risk groups, we used the log-rank test. Cox regression was used for multivariate analysis and the chi-square test was performed for additional estimations. *P*-values < 0.05 were considered significant. Cross-classification tables were used for modality comparison if numbers were considered sufficient and relevant to present interrelationships.

## Results

In total, 111 patients (78 female, 33 male patients) met the inclusion criteria and were eligible for evaluation. Median age at primary diagnosis was 52 years (range 21–82 years) and at initiation of radiotherapy, the median age was 56 years (range 22–82 years). Detailed patient and treatment characteristics are shown in Table 1. In 81% of patients, radiotherapy planning was supported by PET/CT or PET/MRI imaging. Median follow-up was 3.3 years (range 0.3–10.2 years) and the 2-year and 4-year local control rates were 97.6 and 88.7%.

**Table 1:** Patient and treatment characteristics

WHO grading *(n, %)*		
WHO grade I	34	31%
WHO grade II	28	25%
Unknown	49	44%
Initial treatment *(n, %)*		
Primary radiotherapy	49	44%
Initial resection, adjuvant radiotherapy	15	14%
Salvage re-resection, adjuvant radiotherapy	9	8%
Salvage radiotherapy	38	34%
Resection status *(n, %)*		
Gross total resection	29	26%
Subtotal resection	26	23%
Unknown	7	6%
Radiotherapy planning *(n, %)*		
PET-based treatment	90	81%
No PET available	21	19%
Localization *(n, %)*		
Optic nerve sheath / skull base meningiomas	72	65%
Others	39	35%

Abbreviations: *WHO* World Health Organization, *PET* positron emission tomography

**Fig. 2:**
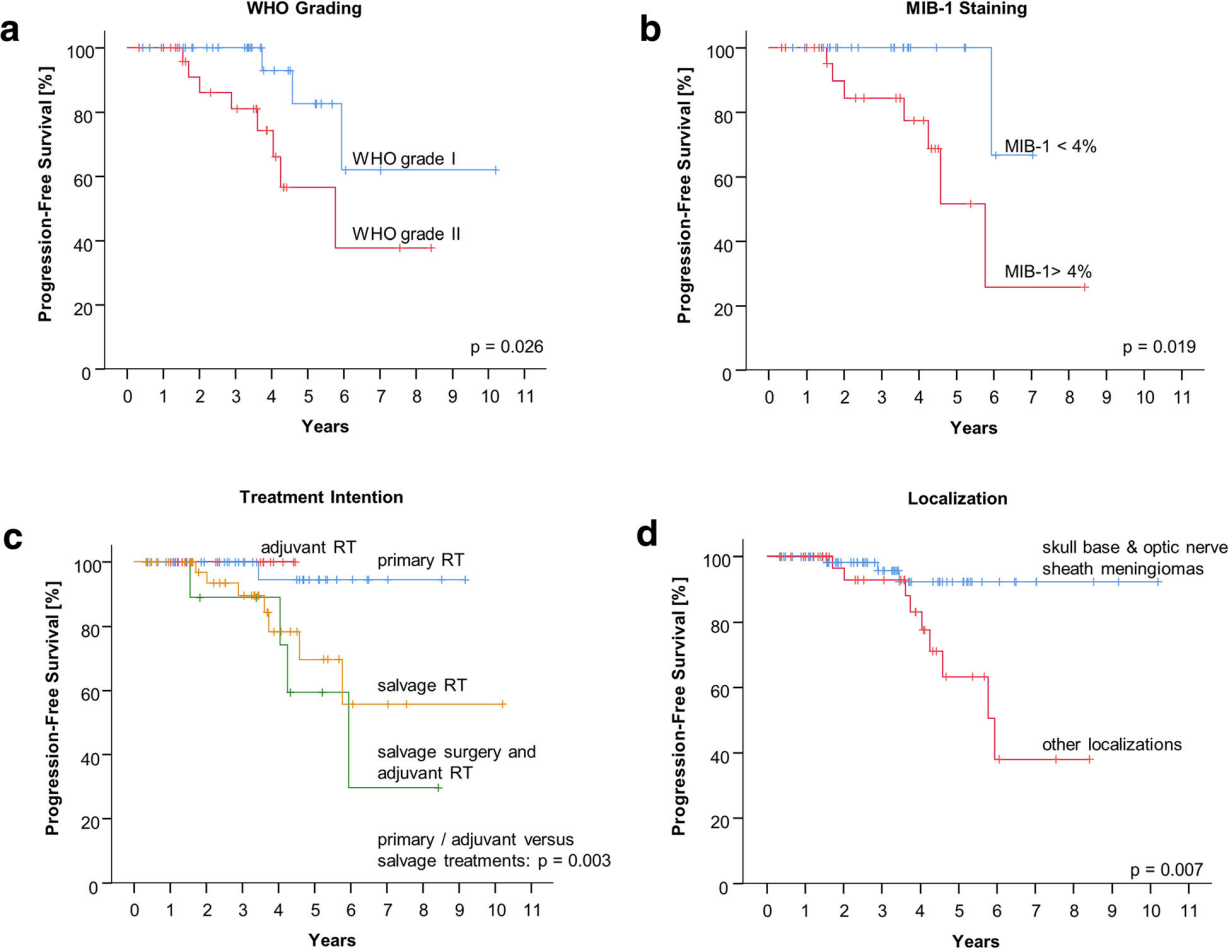
Kaplan-Meier curves for progression-free survival in regard to **a**) World Health Organisation (WHO) grading; **b**) Molecular Immunology Borstel (MIB-1) immunohistochemistry (groups were separated by the median); **c**) intention of radiotherapy (RT) treatment (primary radiotherapy vs. adjuvant radiotherapy vs. salvage surgery and subsequent adjuvant radiation vs. salvage radiotherapy; the *p*-value is related to the primary and adjuvant versus the salvage groups); **d**) localization (skull base and optic nerve sheath meningiomas versus other locations)

In our irradiated cohort, in univariate analysis we found significant differences of PFS stratified by WHO grade I versus WHO grade II meningiomas (*p* = 0.026), estimated MIB-1 staining below 4% (*p* = 0.019), primary or adjuvant radiotherapy versus salvage treatment (*p* = 0.003) and the localization (in favour of skull base and optic nerve sheath meningiomas compared to other localizations, *p* = 0.007). The respective Kaplan-Meier curves are visualized in Fig. 2. In multivariate analysis, none of the univariate prognostic factors remained significant (data not shown). We did not find significant differences in PFS regarding the resection status (subtotal versus gross total resection, *p* = 0.184, data not shown).

**Fig. 3:**
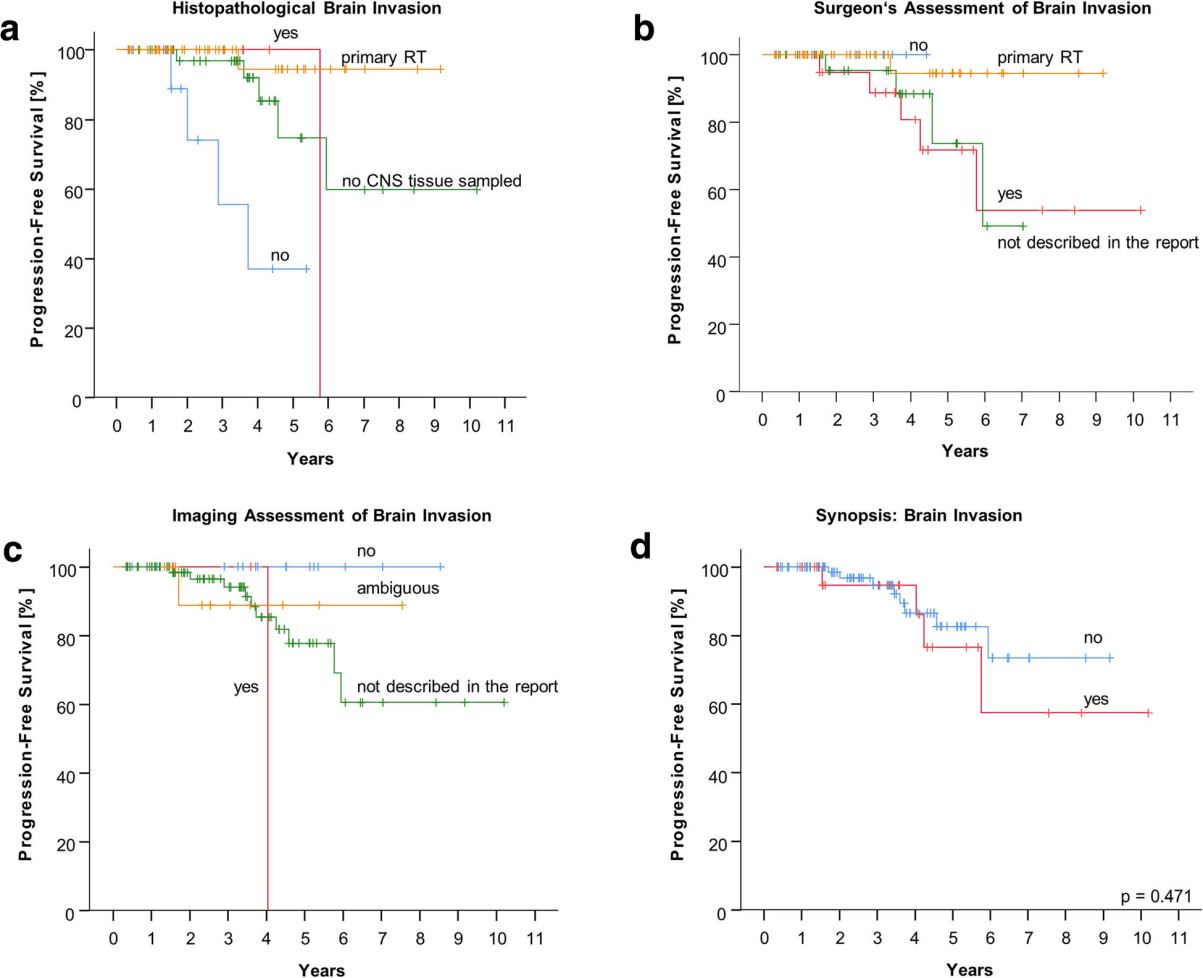
Progression-free survival curves for brain invasion according to **a**) the histopathologic report; **b**) the operative report; **c**) imaging reports and **d**) in synopsis evaluation

Kaplan-Meier plots in regard to brain invasion are shown in Fig. 3. Histopathological, surgical and image-based investigations are illustrated separately. Respective numbers of assessable reports are presented in the Additional file 1: Figure S1. The synopsis (*n* = 111) was plotted individually for clarification.

Patients with histopathologically proven brain invasion (*n* = 9) showed a trend towards improved local control compared to patients without brain invasion (*n* = 12) (Fig. 3a, *p* = 0.062). Considering these subgroups, the patients with histologically proven brain invasive meningiomas were more likely to receive adjuvant radiotherapy (*n* = 6) rather then salvage treatment (*n* = 3). In contrast, patients without brain invasion in histopathology mainly received salvage radiotherapy (*n* = 7) rather then adjuvant treatment (*n* = 3) or adjuvant treatment after salvage surgery (*n* = 2).

In seven patients, brain invasion was explicitly described in the operative report, but no CNS tissue was found according to the histopathology report. In cases where both reports were available, the surgeon’s description of brain invasion was considerably discrepant to histopathologic findings as shown in Fig. 3a/b and the cross-classification table (Additional file 1: Figure S3a, *p* = 0.205). If brain invasion was explicitly considered absent in operative (*n* = 6) or imaging reports (*n* = 16), no event was recorded during follow-up (Fig. 3b/c). In synopsis of the different investigation modalities, brain invasion had no significant influence on PFS in our irradiated cohort (Fig. 3d, *p* = 0.471).

Bone invasion was suspected in 22 patients according to the operative reports but bone tissue was only found in ten histopathologic samples. In eight specimens, bone invasion was confirmed. In one patient bone invasion was suspected by the surgeon but not confirmed histopathologically, and in one case, the operative report is missing but bone involvement was reported in histopathology. Of the patients with pathologically proven bone invasion, only one received adjuvant radiotherapy whilst eight patients were irradiated by means of salvage treatment for recurrence.

The surgical and imaging assessment of bone invasion were concordant in the majority of patients. In 25 patients both reports were assessable. In these, 22 findings were concordant and three were discrepant (Additional file 1: Figure S3b, *p* = 0.001).

**Fig. 4:**
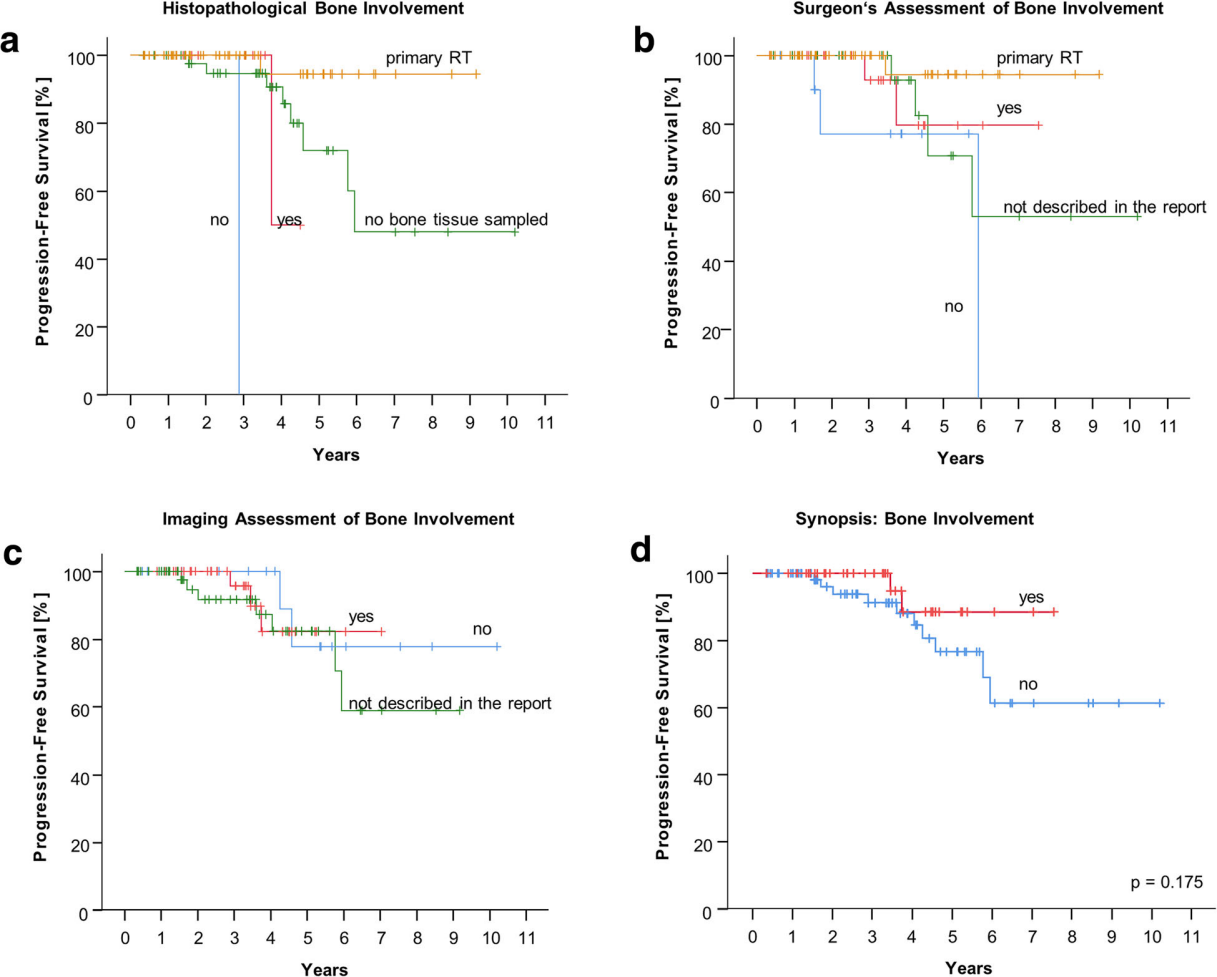
Kaplan-Meier plot for progression-free survival considering bone invasion by **a**) the histopathologic report; **b**) the operative report; **c**) imaging reports and **d**) by synopsis

Figure 4 shows the PFS curves of pathological, surgical and image-based considerations of bone invasion and the synopsis judgement. The patient with negative pathologic report considering bone invasion had tumour recurrence, whilst only one of the pathologically proven bone invasive meningiomas recurred after radiotherapy (Fig. 4a; *p*-value not estimated due to small numbers). Operative and image-based findings did not show significantly different PFS regarding the groups with suspected versus non-suspected bone invasion (Fig. 4b, *p* = 0.207 and 4c, *p* = 0.804). The synopsis consideration also did not differentiate prognosis according to bone invasion (Fig. 4d, *p* = 0.175).

## Discussion

In our cohort of meningioma patients treated with either primary, adjuvant or salvage radiotherapy, several previously described prognostic factors could be confirmed. WHO grading, MIB-1 proliferation activity, initial versus salvage treatment and the localization showed significant prognostic values in univariate analysis. These prognostic factors are well in line with several previous reports [15–17]. However, better prognosis of skull base and optic nerve sheath meningiomas in our study appears contrary to other publications. Convexity meningiomas were reported to perform better than skull base (or generally other) meningiomas due to superior surgical accessibility [16, 18]. This previously proposed prognostic influence of the localization might have been partly overcome by recent surgical techniques or changes in management by elected partial, maximal-safe resections and adjuvant radiotherapy [2]. In spite of potentially diverse genetic and biogical features of skull base and convexity meningiomas, our surprisingly favourable results of skull base or optic nerve sheath meningiomas might be mainly explained by the selection bias of primary irradiated patients, and we presume our significant prognostic factors to be interrelated. As tumours with difficult accessibility are predominantly treated with primary radiotherapy, mostly benign histologic subtypes can be assumed in this subgroup. Convexity meningiomas are more likely to be resected a priori. Those patients typically receive radiotherapy only in case of risk factors or recurrence (negative selection). Therefore, in our cohort, the localization, initial versus salvage radiotherapy, WHO grading and MIB-1 index are supposed to be related. Thus, it appears intuitive that in multivariate analysis none of the prognostic factors remained significant independently.

We decided intentionally not to re-evaluate the histopathologic or imaging reports as the therapeutic decisions in this retrospective study were based on the original reports and not on a potential re-evaluation. The intention was to retrace the daily routine setting without extensive sampling or specifically focused reports. Not all operative or imaging reports commented on brain or bone invasion in spite of potential evaluability, and relevant information could have been missed. Furthermore, MIB-1 staining was solely estimated for the routine reports by different observers, and no exact single-observer re-evaluation was conducted. These considerations might be a benefit (i.e. real setting) and limitation (i.e. potential of some incorrect assessments) of the study at the same time. Further limitations imply the retrospective character and diverse clinical constellations in regard to initial treatment and patient’s performance. Furthermore, image-based consideration of brain invasion is limited to indirect hints such as massive edema or lack of invasion criteria. Besides, some subgroup analyses were limited by small numbers.

Bone or brain invasion did not arise as negative prognostic factors for PFS in synopsis of investigated aspects including pathology, surgery and imaging in our irradiated cohort. According to Perry et al. (1997), judgement of brain invasion implies several pitfalls [6]. First, in many meningioma samples, CNS parenchyma cannot be found and therefore brain invasion cannot be judged due to (intentional) surgical or pathological undersampling. In line with this concern, in several cases of our study, CNS tissue was not available for assessment.

The sampling issues were addressed recently as extensive histopathologic sampling could improve invasion assessability [9]. Other groups did not differentiate between assessable and unassessable specimens, but diagnosed brain invasion in samples without CNS tissue as absent [10]. For pragmatic reasons this might be legitimate and probably reflects the daily practice and interpretation of reports in the clinical setting. However, by different definitions of brain invasion, the controversy of data interpretation about the prognostic value of brain invasion is even aggravated.

Sun et al. (2016) reported on patients who received adjuvant or salvage radiotherapy for meningiomas [19]. Brain invasion was found to be a negative prognostic factor for PFS in both assessable samples and unassessable samples, if those were considered as negative. In contrast, our data indicate that even in assessable samples, the predictive value of brain invasion might be limited. Histopathologically proven brain invasion showed even a trend towards improved PFS compared to patients where brain invasion was explicitly absent. This might be explained by the relevant rate of adjuvant radiotherapy treatment in our patients with histopathologically confirmed brain invasion. This intensified treatment at primary diagnosis could have prevented tumour progression or recurrence. However, these findings have to be interpreted with caution due to the limited patient number in the subgroup and need to be investigated in bigger cohorts.

As a second pitfall, divergent assessments of surgeons and pathologists regarding gross and microscopic findings of brain invasion have been described before and are difficult to interpret [6]. These challenges are in line with our data of relevant discrepancies between surgical findings and pathologic reports. A recent review suggests that a considerable tissue fraction might not be sent to pathology due to intraoperative suction, or it might not be assessable due to subtotal resection [20]. Therefore, the authors recommend sampling of different surface sites and to explicitly provide clinical information to the neuropathologist. This issue is replicated in our data. In seven patients brain invasion was suspected during surgery but no CNS tissue was found in histopathology.

Undersampling cannot be ruled out when using only one modality to judge brain invasion, which we tried to address via synopsis evaluation. For the synopsis we used a ranking including pathological, surgical and image-based information and considered plausibility and time-relation to radiotherapy. Patients without any reliable information about brain invasion were considered as non-invaded. Therefore, all 111 patients could be classified. Synopsis-based consideration of brain invasion did not show a significant influence on PFS.

Interestingly, if the surgeon or the radiologist did exclude brain invasion explicitly, no local failure was recorded in these subgroups. Even though the number of respective patients was rather small, this information might give some indication of improved PFS.

In conclusion, the investigation of brain invasion did not show conclusive results considering PFS in our cohort and might have limited prognostic value in patients undergoing radiotherapy.

Several groups reported on bone involvement as a negative prognostic factor in atypical meningiomas [5, 21, 22]. The issue of different modalities to investigate bone involvement has been addressed before. Pieper et al. (1999) found a distinct association between radiographic evidence and histological validation of involved bony areas [23]. In line with these findings, if bone involvement was described in the operative report, these findings correlated well with imaging and histopathologic evaluations in our study. The single discrepant case with negative pathology might have been due to limited tissue and difficult differentiation between invasion and tumour metaplasia. However, in a relevant number of cases, no bone tissue was assessable for histopathology as, commonly, bone tissue is not routinely sent to pathology in spite of macroscopically visible invasion. The discrepancy between surgically assessed bone involvement and lack of bone tissue in histopathology has previously been observed [5]. The authors suggested a causal relationship of increased dural cauterization and reduced surgical aggressiveness of bone treatment. As a relevant number of local recurrences in case of bone involvement was found in this surgically treated cohort (atypical meningiomas; adjuvant radiotherapy was applied in 23% of cases), the authors speculated on residual tumour cells and discussed the potential benefit of adjuvant radiotherapy including the involved bony regions. In line with these recommendations, in our irradiated patients, bone involvement did not occur as a negative prognostic factor for PFS. Of note, PET imaging has been reported as having higher sensitivity and as being able to maintain specificity compared to MRI in detecting bone invasion [24] and seems to support target volume delineation [11]. However, radiation oncologists have to be aware of the limits of PET imaging with possible overestimation of tumor volumes due to thresholding and underestimate tumor volumes in small volume disease such as dural tails due to partial volume effects. Even though imperfect registrations and resolution issues need to be considered, improved detection of bony involvement by PET imaging might have supported treatment planning and local control in our cohort.

## Conclusions

Brain and bone invasion did not conclusively influence PFS in our irradiated cohort. The potentially negative prognostic impact of brain or bone invasion might have been attenuated by elaborate radiation techniques (IMRT) and the support of advanced diagnostic imaging (PET/CT, PET/MRI). Considerations and assessability of brain or bone invasion can differ notably between diagnostic modalities. This might be addressed by intensified communication between specialties. Furthermore, we recommend a particular integrative synopsis judgement of histopathological, surgical and imaging information as considerable implications occur if invasion is determined.

## Additional file

Additional file 1: **Figure S1.** Overview of assessable reports (‘yes’ or ‘no’) in regard to brain invasion and available, overlapping reports of the different modalities. **Figure S2.** Assessable reports (‘yes’ or ‘no’) in regard to bone involvement and the overlap of the different modalities. **Figure S3.** Cross-classification tables for a) the surgeon’s description of brain invasion and corresponding histopathologic findings and b) operative reports considering bone invasion and associated imaging-based findings. Only assessable and distinct results (‘yes’ and ‘no’) were considered. (PDF 122 kb)

## Data Availability

The datasets analysed during the current study are available from the corresponding author on reasonable request.

## Abbreviations

CNS: Central nervous system
CT: Computed tomography
CTV: Clinical target volume
GTV: Gross tumour volume
IMRT: Intensity-modulated radiotherapy
MIB-1: Molecular immunology borstel
MRI: Magnet resonance imaging
PET: Positron-emission tomography
PET/CT: Positron-emission tomography combined with computed tomography
PET/MRI: Positron-emission tomography combined with magnet resonance imaging
PFS: Progression-free survival
PTV: Planning target volume
WHO: World Health Organization
