# Supplementary material for: Integrative assessment of brain and bone invasion in meningioma patients
Source: Radiat Oncol. 2019 Jul 29;14:132. doi: 10.1186/s13014-019-1341-x (PMC6664715; doi:10.1186/s13014-019-1341-x)
Supplement: Supplementary file 1 — Figure S1. Overview of assessable reports (‘yes’ or ‘no’) in regard to brain invasion and available, overlapping reports of the different modalities. Figure S2. Assessable reports (‘yes’ or ‘no’) in regard to bone involvement and the overlap of the different modalities. Figure S3. Cross-classification tables for a) the surgeon’s description of brain invasion and corresponding histopathologic findings and b) operative reports considering bone invasion and associated imaging-based findings. Only assessable and distinct results (‘yes’ and ‘no’) were considered. (PDF 122 kb) [file 13014_2019_1341_MOESM1_ESM.pdf]

Supplemental Figure 1

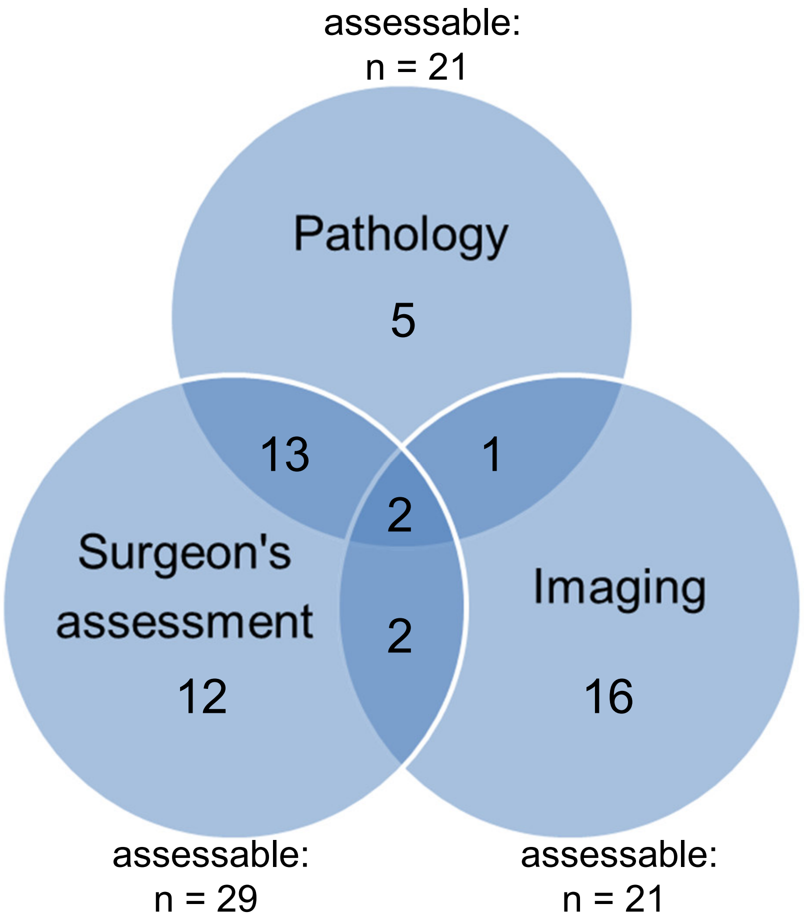

Brain invasion: assessable reports (‚yes‘ or ‚no‘) according to the modalities.

Overview of assessable reports (‘yes’ or ‘no’) in regard to brain invasion and available, overlapping reports of the different modalities

Supplemental Figure 2

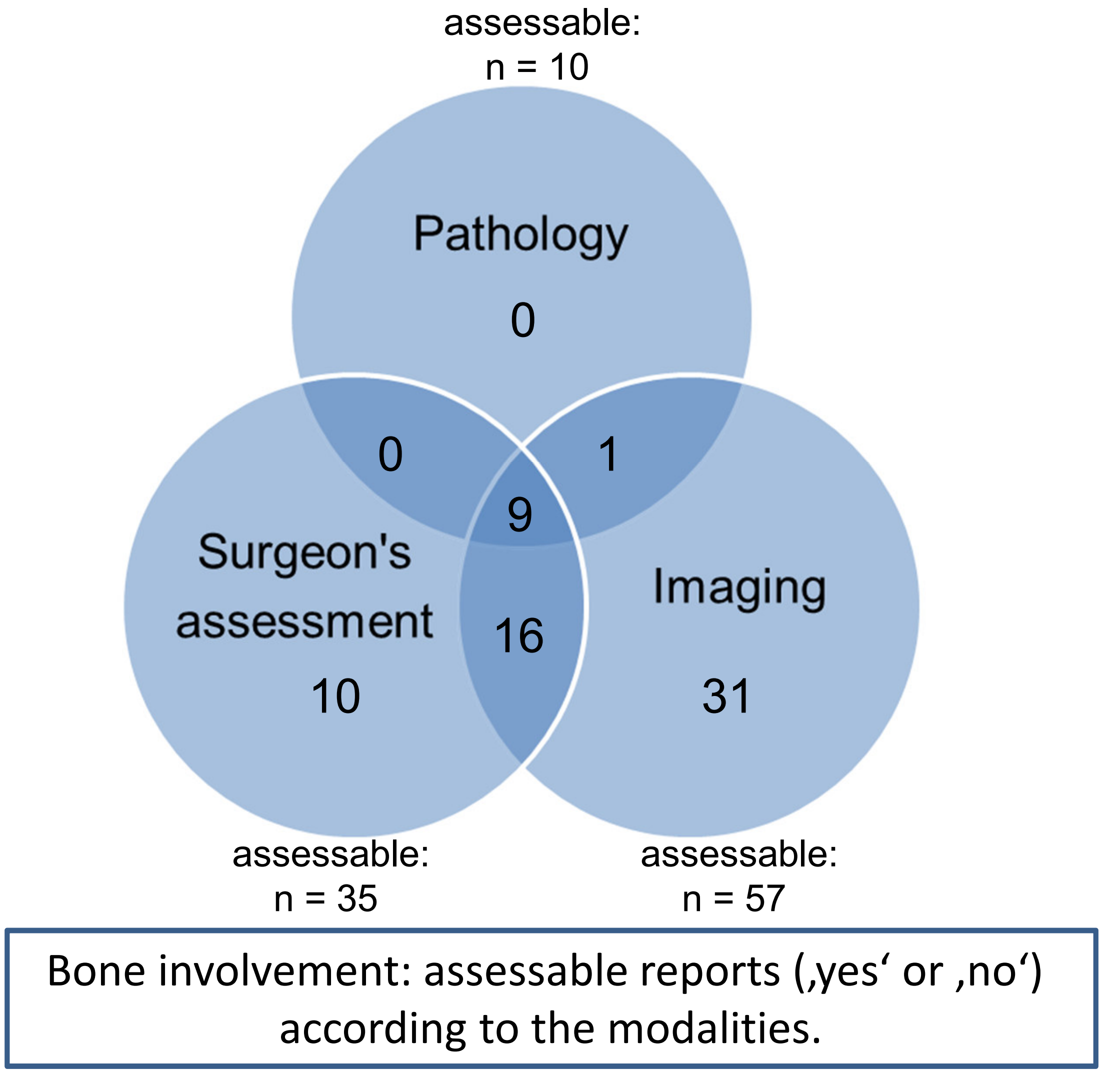

Assessable reports (‘yes’ or ‘no’) in regard to bone involvement and the overlap of the different modalities

Supplemental Figure 3

a		Surgeon's assessment of brain invasion		
		No	Yes	
Pathologically proven brain invasion	No	1	5	6
	Yes	0	9	9
		1	14	

p-value = 0.205

b		Surgeon's assessment of bone involvement		
		No	Yes	
Imaging assessment of bone involvement	No	4	2	6
	Yes	1	18	19
		5	20	

p-value = 0.001

Cross-classification tables for a) the surgeon's description of brain invasion and corresponding histopathologic findings and b) operative reports considering bone invasion and associated imaging-based findings. Only assessable and distinct results ('yes' and 'no') were considered
